# Progress in Evaluation of Deep Artificial Defects from Sweep-Frequency Eddy-Current Testing Signals

**DOI:** 10.3390/s23136085

**Published:** 2023-07-01

**Authors:** Milan Smetana, Daniela Gombarska, Zuzana Psenakova

**Affiliations:** Department of Electromagnetic and Biomedical Engineering, Faculty of Electrical Engineering and Information Technology, University of Zilina, Univerzitna 8215/1, 010 26 Zilina, Slovakia; daniela.gombarska@feit.uniza.sk (D.G.); zuzana.psenakova@feit.uniza.sk (Z.P.)

**Keywords:** electromagnetic non-destructive evaluation, sweep-frequency eddy-current testing, material defect, austenitic stainless steel, multi-point sensing

## Abstract

The article discusses the practical application of the method of electromagnetic non-destructive investigation of austenitic materials. To identify and evaluate deep artificial defects, the sweep-frequency eddy current method with harmonic excitation is used. The objects of interest are the surface electric-discharged machined notches, with a defined geometry, fabricated in a plate with a thickness of 30 mm. An innovative eddy current probe with a separate excitation and detection circuit is used for the investigation. The achieved results clearly demonstrate the robustness and potential of the method, especially for deep defects in thick material. By using the fifth probe in connection with the frequency sweeping of eddy currents, it is possible to reliably detect artificial defects up to 24 ± 0.5 mm deep by using low-frequency excitation signals. An important fact is that the measuring probe does not have to be placed directly above the examined defect. The experimental results achieved are presented and discussed in this paper. The conducted study can serve, for example, as an input database of defect signals with a defined geometry to increase the convergence of learning networks and for the prediction of the geometry of real (fatigue and stress-corrosion) defects.

## 1. Introduction

In the field of eddy-current non-destructive evaluation for defects in conductive materials, some noteworthy advances have been reported in recent years. The identification and characterization of flaws in conductive materials have significantly advanced thanks to the creation of advanced probes, multi-frequency excitation, signal processing methods, electromagnetic modelling, and simulation. These modern trends have improved the safety and reliability of essential components across numerous industries, in addition to improving inspection capabilities. Eddy-current non-destructive evaluations are predicted to play an increasingly important role in assuring the integrity of conductive materials in the future as research and technology continue to advance. Some of the newest developments for thick plate inspection and buried defect evaluation are summarized in this part.

The design of sophisticated probes capable of identifying and characterizing buried or deep flaws is an important trend in eddy-current NDEs. Traditional surface probes are insufficient for examining materials with subsurface flaws due to their restricted penetrating capabilities. Novel probe designs, like multi-frequency, multi-coil, and differential coil combinations, have arisen to get around this restriction. These probes have increased sensitivity and penetration depth, making it possible to find and assess flaws at larger depths. For the inspection of planar conducting plates, Chady and Grochowalski presented an eddy current transducer with spinning permanent magnets. Experimenting on thick aluminium samples with notches at various depths demonstrated the transducer’s viability; ability to identify defects at considerable depths; and advantage of lack of excitation coils, possibly allowing for its operation in hazardous environments [1].

Multi-frequency excitation methods have become more popular recently in eddy-current non-destructive evaluations for deep or buried problems. It is feasible to distinguish signals from flaws at varying depths within the material by using a variety of frequencies. With this method, it is possible to better classify defects, estimate the depth of defects, and differentiate between surface anomalies and more serious problems. When inspecting thick materials or structures with complex geometries, multi-frequency eddy-current non-destructive evaluations have proven to be especially useful. Using tunnelling magnetoresistance (TMR) sensor array, a swept-frequency chirp excitation signal, and a pulse-compression algorithm, Ye et al. [2] have examined ways to improve defect detection and classification capabilities. The effects of using an absolute eddy-current testing (ECT) probe at frequencies close to its electrical resonance were studied by Hughes, Fan, and Dixon [3]. They detected and evaluated the phenomena of defect signal augmentation caused by changes in electrical resonant frequency and report that compared to measurements made at 1 MHz, the phenomenon causes peak signal-to-noise ratios (SNR) to increase by a factor of up to 3.7 at frequencies close to resonance.

A technique for detecting steel plate thinning due to corrosion was created by Tsuyoshi Goda et al. [4], which uses numerous extremely low frequencies and a high-sensitivity magnetic sensor. It uses frequency sweeping, which might take a long time, especially in the very low frequency regions, to obtain a frequency spectrum. In their research, they have created an analytical technique based on the detecting signal’s FFT (Fast Fourier Transform) analysis and a multiple frequency applied magnetic field.

W. Cheng [5] investigated how to measure a metal plate’s thickness using electromagnetic non-destructive testing techniques, specifically swept-frequency eddy-current testing (SFECT) and impedance normalization, without first knowing the material’s precise conductivity and permeability. On paramagnetic and ferromagnetic metal plates, different analytical and experimental tests were carried out. The thickness of non-magnetic metal plates can be calculated by using the extreme values (maximum or minimum) of the normalized SFECT impedance phases that have been discovered. The phase and resistance of very-low-frequency normalized impedance for ferromagnetic metal plates were discovered to be “permeability-independent,” and a conductivity-insensitive function was created. Using this function, the thickness of a ferromagnetic plate was calculated. This study finds that it is likely possible to measure a metal plate’s thickness by SFECT and impedance normalization even without prior knowledge of the conductivity and permeability of the test object.

The efficiency of eddy-current non-destructive evaluation for deep or buried flaws has substantially benefited from developments in signal processing and data analysis methods. Traditional approaches to data interpretation have depended on labour-intensive, erroneous manual analysis. The employment of sophisticated algorithms, machine learning, and artificial intelligence to automate flaw detection and characterisation processes is, nonetheless, a current trend. These methods enable real-time analyses; improve defect recognition; and offer insightful information about the size, shape, and placement of defects.

Using sweep-frequency eddy-current techniques, eddy-current data are gathered at a variety of frequencies. Commercially available equipment can be used to conduct the swept-frequency technique, but it is a challenging and time-consuming method. Given that the depth of penetration changes as a function of frequency, a sweeping-frequency measurement has the advantage of allowing for the acquisition of depth information. Swept frequency measurements are helpful for a variety of tasks, including determining the thickness of conductive coatings on conductive base metals, distinguishing between flaws in base metals and surface coatings, and identifying flaws in different built-up structural layers. They can also aid in determining whether cracking was occurring on the outside skin, the inner skin, or a second layer. The multi-frequency eddy-current testing technique has been empirically shown to increase the signal-to-noise ratio by up to 1100% [6].

SFECT is most frequently employed for layer inspection. Its range of applications includes the simultaneous measurement of substrate conductivity, coating conductivity, and even coating thickness of nonconductive materials. Xu et al. claim that the objective of conductivity evaluations may be accomplished by comparing eddy current testing to a method of parameter measurement that uses apparent conductivity. Coil impedance, plane wave impedance, and their approximate relationships are all evaluated by the authors in their study. They discovered that by comparing the experimental curve of the experimentally achieved equivalent conductivity of coil impedance with the theoretical curves of normalized apparent conductivity of plane wave impedance, all the attributes of the coated plate could be ascertained [7,8,9].

The signals of the to-be-characterized layer are ‘extracted’ from the composite signals while characterizing the multilayered structure. One of the suggested approaches is to characterize the layer of interest by splitting the signals of the various layers by frequency band and using the corresponding signals. A few distinguishing parameters were obtained from the analysis of the simulated signals and the variations of the signal series in the frequency series. High-frequency and low-frequency signals can be used to determine the thickness of the top and bottom layers, respectively. The fluctuations in the signal series in the frequency series are unaffected by the air gap between the two conductive layers. The greatest change in resistance in the frequency series can be utilized to describe the lower layer, even when the conductivity of the plate is unknown [10,11,12,13,14].

Stubendekova et al. [15] dealt with the numerical modelling of the equivalent circuit diagram when using sweep-frequency ECT in COMSOL Multiphysics software. The authors model specific circuitry using the principle of equivalence in electric circuits. The main idea is to use an approach respecting the displacement current density in Maxwell’s equations. In the quasi-stationary EMF approach this component is neglected. The authors point to the fact that defects representing non-conducting volumetric structures can behave as parasitic capacitance. As a result, it is necessary to consider them from the point of view of the theory of electric circuits in the circuit. The validity of this claim and the subsequent effect on the overall response of the system was analysed in the paper.

The main objective of this contribution deals with the issue of direct modifications of the ECT method, namely the SFECT method. The goal is to show the suitability of its use for the detection and identification of deep material defects in manufactured thick AISI 316L plate specimens. Thick conductive plates can be difficult for eddy-current non-destructive inspection due to the skin effect, possible signal attenuation, sensitivity to lift-off variations, and longer inspection time in general. Some of the issues may be addressed by adjusting the inspection technique, probe design, and signal-processing algorithms. Since eddy current inspections rely on the principle of electromagnetic induction, where eddy currents are induced in the material being inspected, the skin effect becomes significant. It refers to the tendency of alternating currents to concentrate near the surface of a conductor, making the currents less effective at penetrating deeper into the material. As a result, the eddy currents induced in a thick conductive plate tend to remain confined near the surface, limiting the depth of inspection. As the eddy currents penetrate the thick conductive plate, they encounter resistance and impedance from the material. This leads to a progressive attenuation of the eddy current signal as it travels through the material. The signal strength decreases rapidly with increasing thickness, making it challenging to detect defects or irregularities located deep within the plate. In addition, the eddy currents induced at different depths can interfere with each other. This interference can result in complex and overlapping signals, making it difficult to interpret the inspection data accurately. Distinguishing between signals from defects and signals generated by the structure itself becomes more challenging, reducing the inspection’s reliability. Eddy current inspection typically requires proximity between the inspection probe and the material surface to achieve accurate results. In the case of a thick conductive plate, maintaining a consistent and precise lift-off (the distance between the probe and the surface) becomes crucial. Any variation in the lift-off distance can significantly affect the inspection sensitivity, leading to false indications or missed detections. Due to the limited penetration depth of eddy currents in a thick conductive plate, a thorough inspection would require performing multiple passes to cover the entire thickness. This increases the inspection time significantly, making it more time-consuming and costly compared to inspecting thinner materials.

For the inspections of used sample of a thick conductive plate with manufactured defects, a special measuring probe was designed and implemented, with a galvanically separated excitation and sensing part. All achieved results were measured using an originally designed and manufactured ECT probe and were obtained experimentally. Numerical modelling of the investigated problem was partially disseminated in the previous works of a wider team of authors. Based on specific outputs of numerical simulations a probe was manufactured, which was subsequently utilised for measurements. The main advantage of the presented approach is the fact that the placement of the probe over the examined material is not necessary directly above the area with the presence of a defect. The implementation of measurements over individual measurement points shows that the SFECT method can reliably detect the presence of defects even in the vicinity of the defect itself. The robustness of this process is especially confirmed by the fact that deep surface defects were successfully investigated, which is evidenced by the achieved results. It should be said that the achieved results will be used as an essential dataset for further processing, where the main goal is the inverse identification of defect geometry. It will therefore be a so-called inverse-approach solution. However, in the presented article, only the results of experiments, i.e., no numerical simulations, are presented.

This paper is organized as follows: the basic theoretical background about the austenitic stainless steels, and the theoretical background and the principle of the method used are introduced in Section 2. Subsequently, the experimental setup is thoroughly presented and described in Section 3. In the following part, the experimental results are presented and discussed in Section 4. The last part, Section 5, brings a summary and evaluation of the achieved results.

## 2. Theoretical Background and Fundamental Principles

The subject of investigation of this paper is austenitic steels with artificially produced defects using the sweep-frequency ECT method. From the theoretical background, all stainless steels, except for the austenitic group, are strongly attracted to a magnet. All austenitic grades have very low magnetic permeabilities and therefore show almost no response to a magnet when in the annealed condition. The situation is, however, far less clear when these steels have been cold worked by wire drawing, rolling, or even centreless grinding, shot blasting, or heavy polishing. After substantial cold working, grade 304 may exhibit quite a strong response to a magnet, whereas grades 310 and 316 will still be almost nonresponsive in most instances. [16]

One of the conventional methods of electromagnetic non-destructive evaluation (eNDE), originating from the electromagnetic induction phenomena, is the eddy current testing (ECT) method. This method is theoretically well-known and widely used in practice. It is suitable for evaluating surface and near-surface defects and is applicable to almost all conductive materials. Many advantages exist, such as high sensitivity, fast scanning, contactless inspection, and versatility, which contribute to its wide utilisation. The basic principle of ECT is relatively simple: it is based on the electromagnetic induction phenomenon (according to Faraday’s law of EM induction). A coil driven with a time-varying current generates the time-varying electromagnetic field (EMF) in its vicinity. Due to the EMF, a time-varying electromotive force is induced in adjacent conductive materials. Therefore, eddy currents flow in the object according to the electromotive force. The EMF generated by eddy currents has the opposite direction in comparison with the exciting EMF generated by a coil. The presence of a defect influences the flow pattern of the induced eddy currents. The impedance of the coil changes due to this fact, giving the means for obtaining information about the material flaw. The ECT problems can be analysed using the quasi-stationary EMF approach. Usually, this approach gives reliable results when the time changes of the EMF are relatively slow, such that the displacement current can be neglected, which applies ***J*** ≫ ∂***D***/∂*t*. This condition applies to conductive materials even at higher frequencies because the conductive current is much higher than the displacement current. The quasi-stationary EMF case is described by Maxwell’s four equations in the following (differential) form:

(1) curl H=J

(2) $$\;\mathrm{curl}\;E=-\frac{\partial B}{\partial t}$$

(3)  div B=0

(4) $$\;\mathrm{div}\;D=\rho_{0}$$

where ***H*** (A/m) is the magnetic field strength, ***E*** (V/m) is the electric field strength, ***B*** (T) is the magnetic flux density, ***D*** (C/m^2^) is the electric flux density, ***J*** (A/m) is the conducting current density, and *ρ*_0_ (C/m^3^) is the volume density of a free charge. The material relations valid for vector quantities of EMF are in the case of linear, homogeneous, and isotropic environments in the following form:

(5) D=εE

(6) B=μH

(7) J=γE.

where *ε* (F/m) is the permittivity, *μ* (H/m) is the magnetic permeability, and *γ* (S/m) is the electrical conductivity of a material. The EMF can be analysed using the potential functions:

(8) B= curl A

(9) $$\mathrm{grad}\;V=-E-\frac{\partial A}{\partial t}$$

(10) div A=0.

where ***A*** (T·m) is the magnetic vector potential, and *V* (V) is the electric scalar potential. The ECT analysis is conducted by both quantities. The solution domain is subdivided into a conducting area *Ω*_1_ and non-conducting area *Ω*_2_. The eddy currents in the conductor are governed by the following equations in:

(11) $$\mathrm{air}\;\mathrm{region}:\;\;\;\;\;\;\;\;\;\;\;\;\;\;\;\;\;\;\;\;\;\;\;\;\nabla^{2}A=0$$

(12) $$\;\mathrm{coil}\;\mathrm{region}:\;\;\;\;\;\;\;\;\;\;\;\;\;\;\;\;\;\;\;\;\;\;\;\;\;\nabla^{2}A=-\;\mu J$$

(13) $$\;\;\;\;\;\mathrm{conductor}\;\mathrm{region}:\;\;\;\;\;\;\;\;\;\;\;\nabla \mu \sigma \left(-\nabla V-j\omega \nabla V\right)=0$$

(14) $$\nabla^{2}A-j\omega A-\mu \sigma \nabla V=0.$$

Sweep-frequency eddy-current techniques involve collecting eddy current data at a wide range of frequencies. The advantage of this measurement is that depth information can be obtained since eddy-current depth of penetration varies as a function of frequency. Sweep-frequency measurements are useful in applications, such as measuring the thickness of conductive coatings on conductive base metal, differentiating between flaws in surface coatings and flaws in the base metal and differentiating between flaws in various layers of a built-up structure. Frequency measurements would make it possible to tell if defects were occurring on the outer skin, the inner skin, or a double layer. The main difference between the SFECT method and the conventional ECT is that, in the case of the method used, the measuring probe is statically placed above the examined surface. The probe does not move during the measurement. The movement is replaced by modulating the excitation signal of the probe, while depending on the type and purpose of its use, the frequency interval must be chosen appropriately [17,18]. During the inspection, two cases can occur: the probe is placed over the location with the present defect, or this location can be predicted. In the second case, the probe is placed over the material randomly. In this case, it is necessary to know the characteristics and behaviour of the useful signals since it is a comparative method. During the measurements, it was found that the presence of artefacts in the detected signals also has a significant impact on the information value of the signals; it is mainly a superimposed signal of the conductive structure itself and the characteristic of the probe itself, which is placed in the air. All these influences need to be eliminated to increase the resistance of the resulting signal. In this study, an approach was used where the measurement points were placed over the region of interest (ROI) in a defined manner [19,20].

## 3. Experimental Set-Up

**Figure 1:**
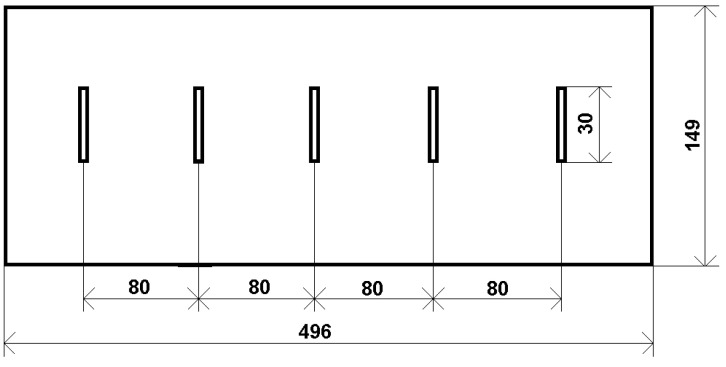
Spatial configuration of the inspected AISI 316L specimen with individual EDM defects.

**Figure 2:**
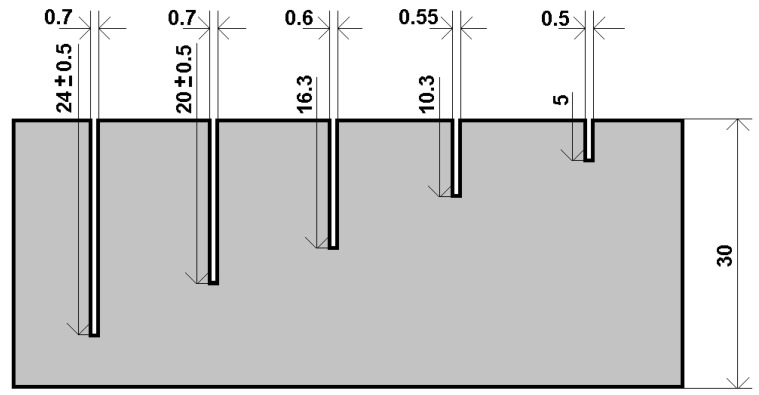
Side view of the specimen with five non-conductive EDM defects with varying in-depth parameters.

For the purposes of this study, a specimen of a conductive plate with thickness *h*_P_ = 30 mm and electromagnetic parameters of AISI 316L (austenitic stainless steel) was utilized. The material had an electrical conductivity of *γ*_P_ = 1.38 MS/m and a relative magnetic permeability of *μ*_rP_ = 1. The specimen contained five non-conductive electric-discharge-machined (EDM) defects with a rectangular (cuboid) shape, as shown in Figure 1. The plate had defects with an average width of *w*_c_ = 0.61 mm, a constant length of *l*_c_ = 30 mm, and their depth *d*_c_ was varying in the range of 5–24 mm with a step of 5 mm, as shown in Figure 2. The electrical conductivity of all defects had a zero value, or it was equal to the air conductivity, i.e., *γ*_D_ = 0 S/m.

The specimen with the defects was manufactured based on precisely defined requirements. The shape, dimensions, and parameters of the defects (conductivity) were clearly defined, based on previous numerical simulations, carried out in software for EMF simulations (Opera, Vector Fields). The realized specimen together with defects and its properties were guaranteed by the manufacturer based on measurements using three methods based on different physical principles (ultrasound testing (UT), radiography testing (RT), and electromagnetic acoustic transducer technique (EMAT)). Because of these facts, the specimen can be considered as an extremely precise pattern, that is, a so-called etalon, Table 1.

**Figure 3:**
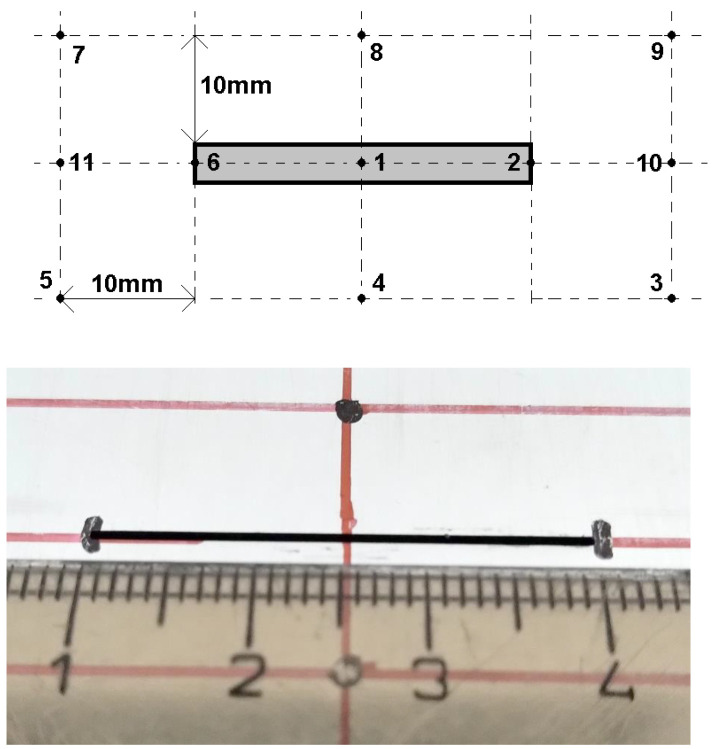
Positioning and numbering of the individual measurement points above the ROI (**top**) and real defect view (**bottom**).

Individual measurement points are located axially and symmetrically concerning the length dimension of the defect. They are chosen to ensure uniform coverage of the area directly above the defect itself and in its vicinity, Figure 3. Based on this distribution, each defect is measured a total of eleven times. The probe is always positioned in such a way that the receiver is located exactly above the sensing point and the excitation coils are positioned parallel to the length dimension of the defect. The lift-off parameter is chosen as lift-off = 1 mm because of the limitations of the probe-design construction (Teflon protective case). In addition to the signals from the measuring points, the background signals must also be considered during the measurement; it is mainly the signal of the measuring probe itself located in the air and the signal of the probe located near the material without the presence of a defect. These signals need to be subtracted from the useful signals of the measuring points in a suitable way. This creates a differential response that represents the resulting desired signal. This procedure must be followed because the ECT method and its modifications are comparative methods. Measuring points number 1, 4, and 8 can be considered as points when the measuring probe is placed across the width of the defect. Measuring points 1, 2, 6, 10, and 11 can be considered as measuring points when the measuring probe is placed across the length of the defect. Overall, the measuring points are distributed in such a way that it is possible to reliably identify the presence of a defect when the probe is in a fixed position above the material under examination.

**Figure 4:**
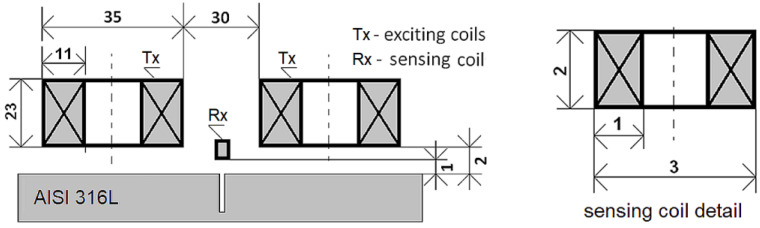
Configuration and geometry of the designed SFECT probe above the material with defect. (All dimensions are in millimetres).

The SFECT probe consists of two exciting coils (transmitter, Tx) that are positioned normally adjacent to the surface of inspected material and apart from each other, as shown in Figure 4.

**Figure 5:**
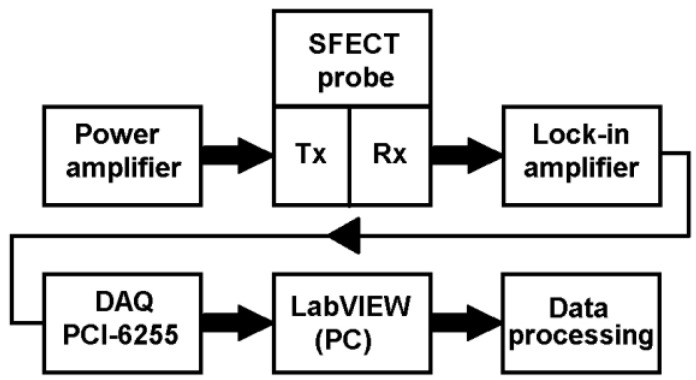
Measuring apparatus connection diagram: flowchart among individual equipment and function blocks.

Two exciting coils with selfinductances of *L*_1_ = 3.10 mH, and *L*_2_ = 3.13 mH are driven by a harmonic current with an effective value of *I* = 1.2 A. The range for frequency sweeping is set as follows: *f* ∈100 Hz; 5 kHz with the discrete step of *f_step_* = 25 Hz. The inductance receiver coil has *N* = 600 number of turns, and it is wound from a copper wire with a diameter of *Ø*_Cu_ = 0.05 mm. The sensing element is positioned in the middle between the exciting coils. The coils are connected in-series but magnetically opposite to decrease the coupling between the exciting system and the sensing coil. The high sensitivity of a pick-up element can be achieved in such cases. The detected signal from the receiver is sent to the input of the Lock-in amplifier (Signal Recovery, model DSP 7280). The signal for the frequency sweeping output is taken from its internal precise oscillator. This signal is fed to the input of the broadband linear power amplifier (Krohn-Hite, model 7500), which directly drives the excitation coils of the probe, Figure 5.

**Figure 6:**
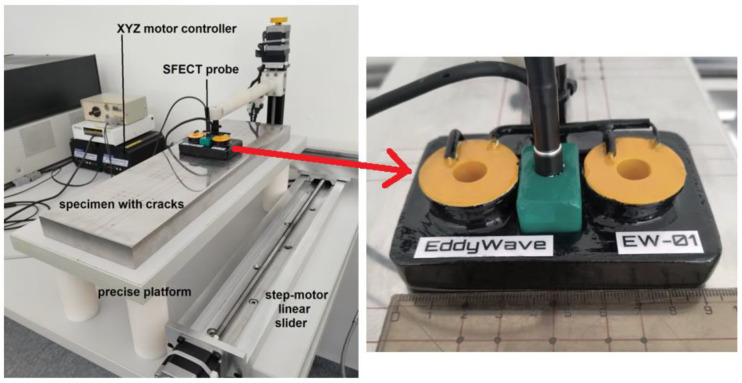
Overall view of the process of electromagnetic non-destructive evaluation using the SFECT method and detail of innovatively designed and realized probe. Workplace: Laboratory of Electromagnetic Non-Destructive Evaluation (DEFECTOLAB), University of Zilina.

The output of the Lock-in amplifier is a decomposed harmonic signal; the real and imaginary parts of the induced voltage are decoded separately. These signals are weighted and normalized by the internal circuits of this device, depending on the current parameter settings (gain, dynamic reserve, buffer, time constant, etc.). They are sent to the peripherals after amplification in analogue form on the output terminals. These two signals are captured using a recording card (Digital Acquisition Card, DAQ, model PCI-6255), which works directly with the LabVIEW (National Instruments) graphic development environment. Measured data are acquired using the data acquisition card with a resolution of 16 bits/channel, 10 ks/s. In this development environment, the program itself is also created for the automated control of the probe’s movement using a PC-controlled linear slider (Figure 6), data recording and collection, and their subsequent processing into the required format. The postprocessing of the data itself, the necessary mathematical operations, and calculations are carried out in MATLAB (by Mathworks) software.

## 4. Experimental Results and Discussion

**Figure 7:**
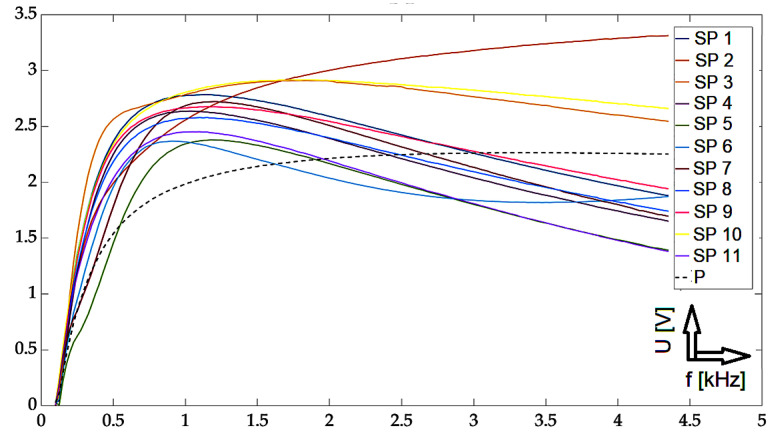
Experimental results: defect #1, absolute value of the receiver-coil-induced voltage on frequency, scanning positions 1–11 and defect-free signal, and subtracted signals.

**Figure 8:**
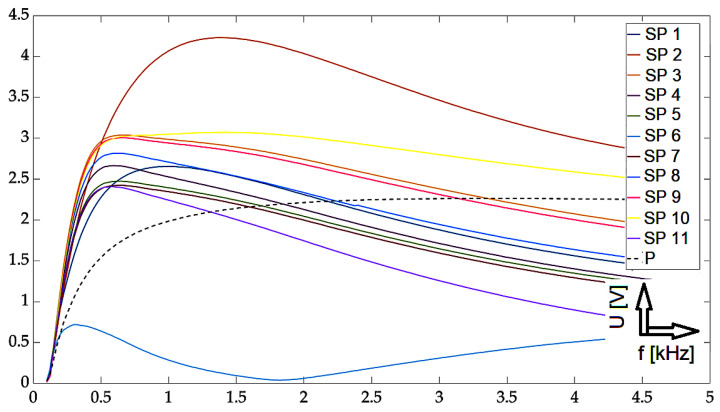
Experimental results: defect #3, absolute value of the receiver-coil-induced voltage on frequency, scanning positions 1–11 and defect-free signal, and subtracted signals.

**Figure 9:**
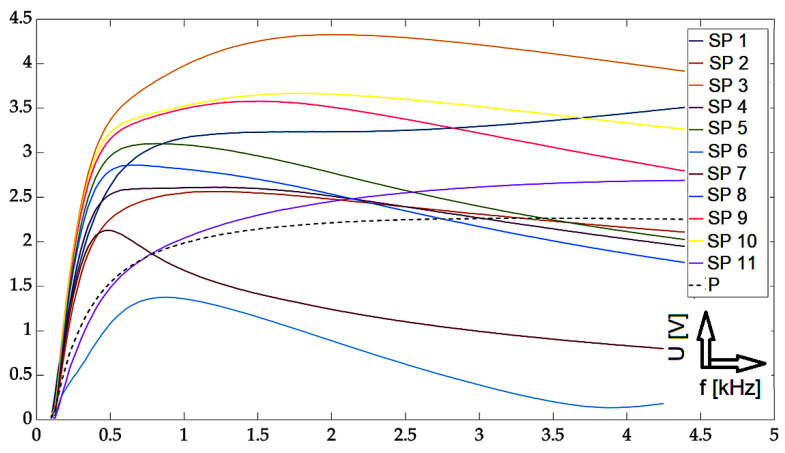
Experimental results: defect #5, absolute value of the receiver-coil-induced voltage on frequency, scanning positions 1–11 and defect-free signal, and subtracted signals.

In this part, individual mathematical formulations are presented which were used to calculate the necessary values for rendering in graphs. At the same time, the results of the implemented experiments are presented here, with the corresponding description and discussion. All measurements were carried out using two approaches: The first one was the so-called near-side approach, in which the measuring probe was placed on the side of the defects. In the second approach, the so-called far-side, the measuring probe was placed on the opposite side of the specimen, meaning the presence of defects was not visually present. Such an approach can simulate a real situation when individual defects are not visually detectable in the examined material. Two different methods are used for the graphical display of the achieved results: the first method was the display of the absolute value of the induced voltage, depending on the frequency. The second method was a representation of the induced voltage of the receiving probe as a decomposed signal in a Gaussian plane. The realized waveforms are in the form of Lissajous figures. The following section presents the achieved results in graphic form. These are the results of measurements on the investigated conductive structure. Figure 7, Figure 8 and Figure 9 show the dependence of the absolute value of the detected signal as a function of frequency. The figures show the results for defects #1, #3 and #5, respectively. For better clarity and readability of the graphs, the waveforms obtained for defects #2 and #4 are not shown. The individual graphs display the waveforms obtained from individual measuring points (scanning point, SP). At the same time, the signal above the specimen without defect (plate signal, P) is also added to the graph. To increase the information value of the waveforms, the following procedure was used to process these measurement results: signals from measurement points 1 to 11 were inserted into a 2D matrix. This procedure is applied to both near-side and far-side measurements. Individual matrices are subtracted from each other, respecting the order of individual measurement points, or their mirroring. This creates a new 2D matrix whose individual elements represent the resulting signals to be displayed.

From the graphs, the character of the signals for the three selected defects can be seen: from the shallowest to the deepest defect. In the selected frequency range, signals that are clearly separated from each other can be identified in individual sections. Depending on the specific location of the measuring probe, these signals can be differentiated by their values from the set of other waveforms. At the same time, the distances among the individual signals increase in the direction with the increasing depth of the defect. This phenomenon is very desirable because it allows for the detection of deeper material defects. Further, it can also be seen that at lower frequencies/initial values of the sweeping interval, the detection ability is very low, which is in accordance with theoretical knowledge. The information value of the detected signals has an information value for frequencies of 500 Hz and above. The localization of the probe is a crucial factor in determining its geometry. For some of the positions, the obtained curves were higher in amplitude or lower than the signal for the material itself without the presence of a defect. By comparing these curves with all others, it is possible to determine whether there is a defect in the material or not.

**Figure 10:**
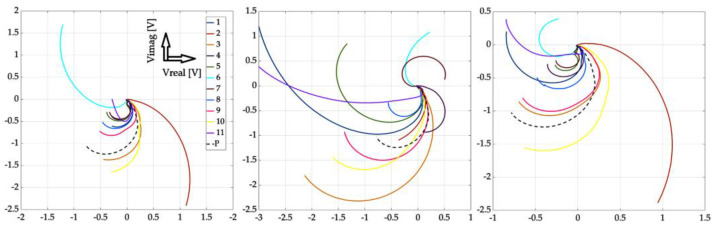
Experimental results (from left to right): defect #1, #3, and #5; near-side approach; decomposed receiver-coil-induced voltage; scanning positions 1–11; and plate signal (P).

**Figure 11:**
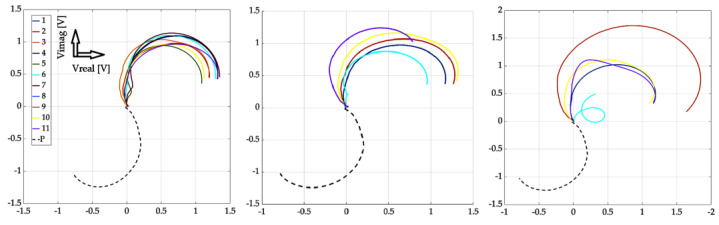
Experimental results (from left to right): defect #1, #3, and #5; far-side approach; decomposed receiver-coil-induced voltage; scanning positions 1–11; and plate signal (P).

**Figure 12:**
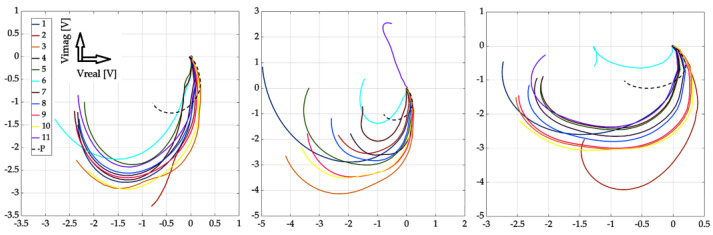
Experimental results (from left to right): defect #1, #3, and #5; subtracted responses; decomposed receiver-coil-induced voltage; scanning positions 1–11; and plate signal (P).

Figure 10, Figure 11 and Figure 12 show the achieved results in the form of a display in a complex plane, i.e., in the form of the real and imaginary part of the induced voltage of the detection coil. Figure 10 shows the waveforms for investigation using the near-side approach, Figure 11 shows using the far-side approach, and Figure 12 shows using the subtraction of individual 2D signal matrices for both approaches simultaneously. The colour-coded waveforms correspond to the individual measurement points, including the display of the signal for the defect-free plate.

From the mentioned curves, it can be concluded that the resolution of individual signals increased as the depth of the EDM defects increased, which was the aim of the investigation. At the same time, we can clearly say whether or not there is a defect in the given place of examination. The characteristics of the signals obtained from the examined plate without the presence of a defect are clearly different from all the other waveforms. Therefore, it can be argued that by processing such signals, the presence of a defect in the examined sample can be clearly distinguished. A very interesting result is the fact that when using the far-side approach, the situation is even clearer. The convergence of individual curves for individual scanning points means that the probe can be placed at any point when using the given scanning method. The resulting information value is comparable among the individual curves.

**Figure 13:**
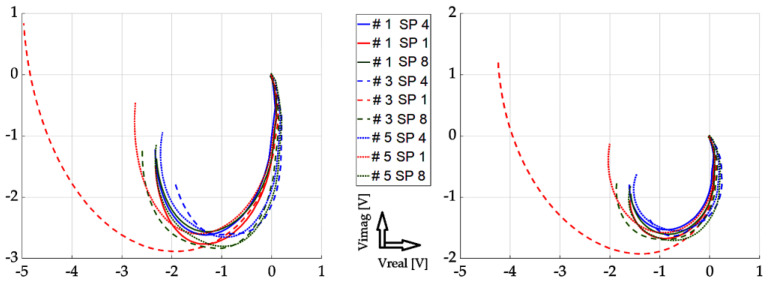
Experimental results: defect #1, #3, and #5; subtracted responses; decomposed receiver-coil-induced voltage; scanning points across the width parameter (**left**); and across the length parameter (**right**).

**Figure 14:**
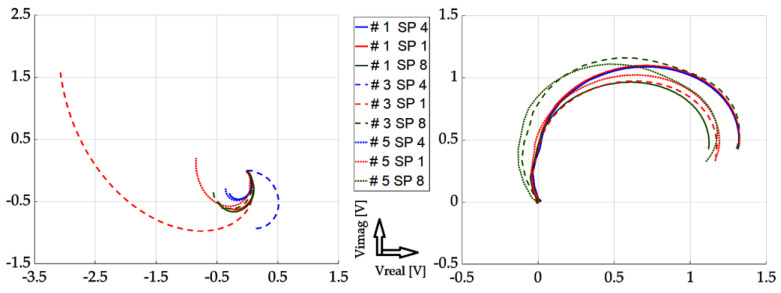
Experimental results: defect #1, #3, and #5; near-side approach (**left**) and far-side approach (**right**); decomposed receiver-coil-induced voltage; and scanning positions across the defect width.

**Figure 15:**
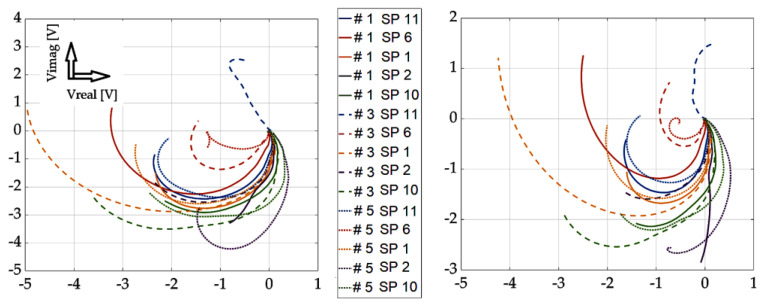
Experimental results: defect #1, #3, and #5; subtracted responses; decomposed receiver-coil-induced voltage; scanning positions across the defect length (**left**); and across the defect width (**right**).

**Figure 16:**
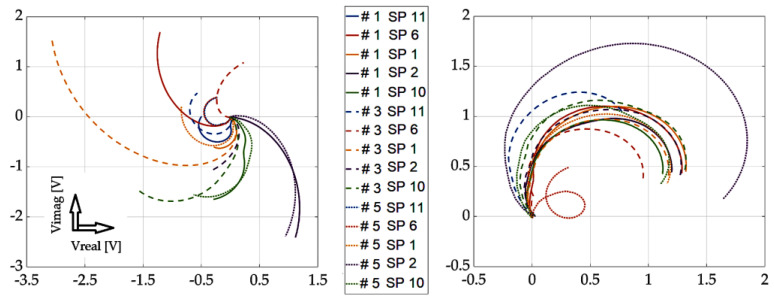
Experimental results: defect #1, #3, and #5; near-side approach (**left**) and far-side approach (**right**); decomposed receiver-coil-induced voltage; and scanning positions across the defect length.

Figure 13, Figure 14, Figure 15 and Figure 16 show the waveforms from selected measurement points and scan approaches related to probe placement, i.e., across the defect width and the defect length. These signals are shown for three specific defects for better clarity of the results. The results show that the SFECT method is less sensitive to the “scanning” method, or the placement of the probe over the area of interest. When using the classical approach, if the probe moves parallel to the length dimension of the defect, it is very difficult to identify the defect. Using the mentioned method of probe placement and the SFECT method, it was shown that this aspect is not crucial, and thus, the defect can be identified using near-side, far-side, or the mutual subtraction of these two scanning methods. The robustness of the entire methodology also lies in the use of the low-frequency excitation signals of the probe.

If there is a request to assess the geometry (especially the depth) of defects, it is desirable to place the probe directly above the place where it is present. This is evidenced by the previous graphs, where the probe was placed directly in the centre and on the edges of the defect, separately. The robustness of the obtained results lies in an innovative approach: the mutual subtraction of value matrices for both scanning methods and mirror-rotated data matrices. If the sample does not contain a defect, the resulting signal is zero (beginning of the Gaussian plane). As a result of the different geometry of the examined defects, there is a deviation of the signal (see the relevant graphs). As the depth of the defect increases, the radius of the described circle of the curve increases, and the spacing between the curved characteristics also increases. By analysing and processing these signals, it is possible to gather information about the investigated inhomogeneities in the material. As part of the overall evaluation of the achieved results, the following conclusions can be stated: the reliability of the detection and identification of the investigated defects lies mainly in the use of the low-frequency excitation signals of the probe, even more so with the appropriate distribution of fixed measuring positions and with the appropriate mathematical processing of the detected signals. Through the simultaneous use of these three attributes, valuable results were obtained, which can be interpreted as the successful detection of deep artificial EDM defects in the investigated austenitic material.

## 5. Conclusions

eNDE is a powerful tool for the investigation of various types of materials and biomaterials. In this contribution, the use of the SFECT method as a robust tool in the detection and identification of deep surface defects located in an electrically conductive structure was addressed. The subject of investigation was artificially produced EDM defects of a defined shape with varying depths. The thickness of the austenitic steel plate was 30 mm, while the deepest of the defects had a depth of 24 mm. By using the SFECT method in combination with a newly designed and implemented probe, measurements of individual defects were made. The measuring positions were selected in a defined manner and placed over the investigated material. Low-frequency harmonic signals, ranging from 100 Hz to 5 kHz, with a step of 25 Hz, were used to excite the probe. A digital lock-in amplifier, based on DSP, was used for signal filtering. The following approaches were used for signal processing: near-side, far-side, and a system of relative reading of these two approaches, at all measurement points. The signals obtained in this way were further mathematically processed by removing background artefacts (the characteristic of the measuring probe and the influence of the investigated material itself). From the achieved results, it can be concluded that the method used brought positive findings and expanded the scientific horizons. This is mainly due to the following facts: all defects were reliably detected from individual measurement points, and the influence of the location of the measuring probe in connection with the mathematical processes of signal processing used did not prove to be critical. In other words, it can be said that defects could be detected even when the position of the probe deviated from the investigated defect. Another valuable result was the use of the superposition of signals from the corresponding measurement points when investigating the near-side and far-side modes. From the achieved results, when the matrices of such signals are subtracted, the information value of the signals increases several times. From the results, it can be seen that the mentioned approach is suitable for identifying deeper defects, specifically with a depth of 10 mm and deeper. At the same time, it is possible to identify the defect when placing the probe above the examined surface, directly above the defect (measuring points 1, 4, 8) but also when placing the other measuring points. In addition, when using the far-side scanning system (simulation of a real case of investigation), the presence of a defect in the material can be clearly detected, which is very desirable.

The authors’ future work will be focused on the use of machine learning for the backward identification of defect geometry, depending on the input database of base signals, using the abovementioned method towards inverse-problem solution.

## Figures and Tables

**Table 1:** Artificial EDM defect geometry: main dimensions according to the manufacturer’s datasheet (Advanced Technology Group, ATG Ltd., Czech Republic).

Defect No.	Width [mm]	Length [mm]	Depth [mm]
#1	0.5	30	5
#2	0.55	30	10.3
#3	0.6	30	16.3
#4	0.7	30	20 ± 0.5
#5	0.7	30	24 ± 0.5

## Data Availability

The data presented in this study are available upon request from the corresponding author.
